# A Rare but Aggressive Malignancy: A Case Report of a Gastrointestinal Neuroectodermal Tumor (GNET)

**DOI:** 10.7759/cureus.41509

**Published:** 2023-07-07

**Authors:** Shahzeb Saeed, Han Grezenko, Lyba Nisar, Abdur Rehman, Amina Riyaz, Daniel E Cook, Muhammad Kamran

**Affiliations:** 1 Internal Medicine, Army Medical College, Islamabad, PAK; 2 Medicine, Guangxi Medical University, Nanning, CHN; 3 Internal Medicine, Quaid-e-Azam Medical College, Bahawalpur, PAK; 4 Surgery, Mayo Hospital, Lahore, PAK; 5 Medical School, Sree Uthradom Thirunal (SUT) Academy of Medical Sciences, Trivandrum, IND; 6 International Medical Graduate, Avalon University School of Medicine, Youngstown, USA; 7 Internal Medicine, Mayo Hospital, Lahore, PAK

**Keywords:** aggressive tumor, malignant neuroectodermal tumor of gastrointestinal tract, gastrointestinal neuroectodermal tumor, malignancy, surgery, neuroendocrine tumor, rare gastric tumor, gnet

## Abstract

Gastrointestinal neuroectodermal tumors (GNETs) are extremely rare and intriguing malignancies originating from neural crest cells in the digestive tract. The digestive tract's neural crest cells can give rise to incredibly unusual and interesting gastrointestinal neuroectodermal tumors (GNETs). GNETs present considerable hurdles in diagnosis and management because of their rarity and varied expression. In this case report, a 45-year-old male patient is described who had signs of GNET, such as exhaustion, weight loss, and abdominal pain. A 7-cm jejunum tumor and related thickening of the gut wall were discovered using imaging investigations. The diagnosis of malignant GNET was confirmed by surgical resection, and adjuvant treatment was given. A recurring tumor required a second surgical procedure despite an initial disease-free period. The report emphasizes the difficulties involved in the diagnosis, treatment, and long-term effects of GNETs. The rarity of GNETs necessitates the development of standardized treatment protocols as well as additional research to enhance diagnostic precision and explore novel therapeutic approaches for this aggressive malignancy.

## Introduction

Malignant gastrointestinal neuroectodermal tumors (GNETs) are exceedingly rare and highly intriguing neoplasms that arise from neural crest cells within the digestive tract. These tumors, characterized by their neuroectodermal origin, present a complex clinical and pathological challenge due to their scarcity and the inherent heterogeneity in their presentation. Although the precise etiology of GNETs remains elusive, their classification as a distinct entity is founded upon their unique histological features and molecular characteristics [1].

GNETs primarily affect adults, with the age at diagnosis typically ranging from the second to the seventh decade of life. Clinical manifestations vary widely and are often nonspecific, encompassing symptoms such as abdominal pain, weight loss, fatigue, and gastrointestinal bleeding. The lack of specific clinical features further complicates the early diagnosis and recognition of GNETs, frequently resulting in delayed or missed diagnoses [2].

Imaging studies play a pivotal role in the evaluation and initial identification of GNETs. Radiologically, these tumors present as intraluminal masses with associated features such as bowel wall thickening, ulceration, and mesenteric lymphadenopathy. However, it is important to note that the radiological appearance of GNETs may mimic other gastrointestinal malignancies, making accurate diagnosis challenging and warranting histopathological confirmation [3].

Histologically, GNETs exhibit a diverse range of patterns, including solid, nested, trabecular, and glandular formations. Immunohistochemistry plays a critical role in establishing the diagnosis, with GNETs typically showing positivity for markers such as S100, neuron-specific enolase (NSE), synaptophysin, and CD56. The expression of these neuroendocrine markers supports the neural crest origin of GNETs and helps distinguish them from other gastrointestinal tumors [4].

Due to their rarity, the management and treatment protocols for GNETs are not well established. The majority of treatment is surgical resection, which aims to completely remove the tumor and may improve patient outcomes. Although its efficacy in GNETs is unknown, adjuvant chemotherapy is frequently used to lower the risk of local recurrence or distant metastasis.

This case study describes a 45-year-old male with GNET symptoms, including the clinical presentation, radiological findings, results of the histological examination, and course of treatment. After surgery and chemotherapy, the patient experienced periods without having any symptoms, but a tumor recurrence necessitated a second surgery. The complexity of detecting, treating, and foreseeing the long-term effects of GNETs is brought home by this instance.

## Case presentation

We present the case of a 45-year-old male who sought medical attention due to a six-month history of abdominal pain, weight loss, and fatigue. The patient reported a significant weight loss of 10 pounds during this period and described the abdominal pain as progressively worsening. He had no notable medical or surgical history and no family history of cancer.

A physical examination revealed a pale and fatigued appearance. Abdominal palpation revealed a soft abdomen without tenderness, palpable masses, or hepatosplenomegaly. Laboratory investigations revealed anemia, with a hemoglobin level of 8.5 g/dL and elevated lactate dehydrogenase (LDH) levels. Other laboratory parameters were within normal ranges. We considered several possible diagnoses, including colon cancer, gastrointestinal neuroendocrine tumor (GNET), gastrointestinal stromal tumor (GIST), and synovial sarcoma.

**Figure 1:**
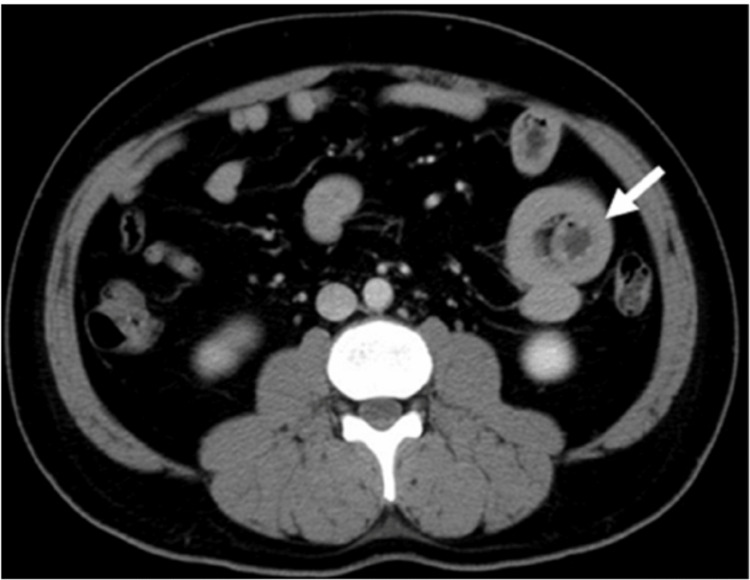
A CT scan of the abdomen showed a 7-cm mass in the jejunum with associated bowel wall thickening (arrow).

To further evaluate the patient's condition, a contrast-enhanced computed tomography (CT) scan of the abdomen was performed (Figure 1), which revealed a substantial 7-cm mass located in the jejunum.

Additionally, the CT scan revealed bowel wall thickening and mesenteric lymphadenopathy, but no evidence of distant metastases was observed. Upper gastrointestinal endoscopy and colonoscopy were subsequently performed, ruling out the presence of any additional lesions.

**Figure 2:**
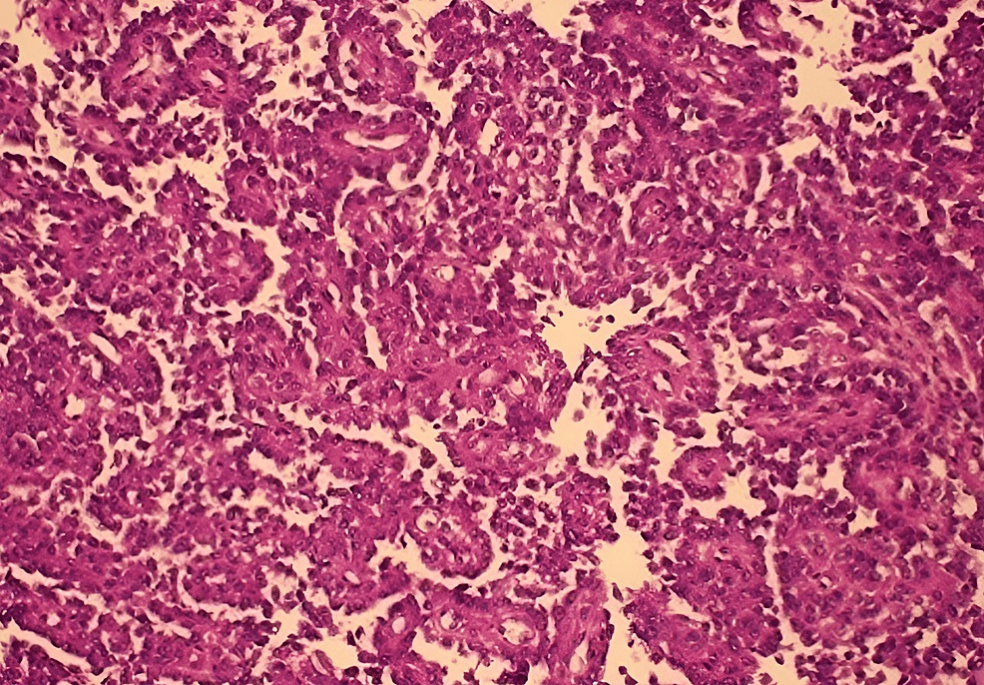
The biopsy showed loosely discohesive epithelioid cells with eosinophilic to clear cytoplasm, round to ovoid nuclei, and prominent nucleoli.

Considering the clinical and radiological findings, the decision was made to proceed with surgical intervention. The patient underwent surgical resection, which involved an extended bowel resection to completely excise the identified mass. Histopathological examination of the resected specimen established the diagnosis of a malignant GNET (Figure 2).

**Figure 3:**
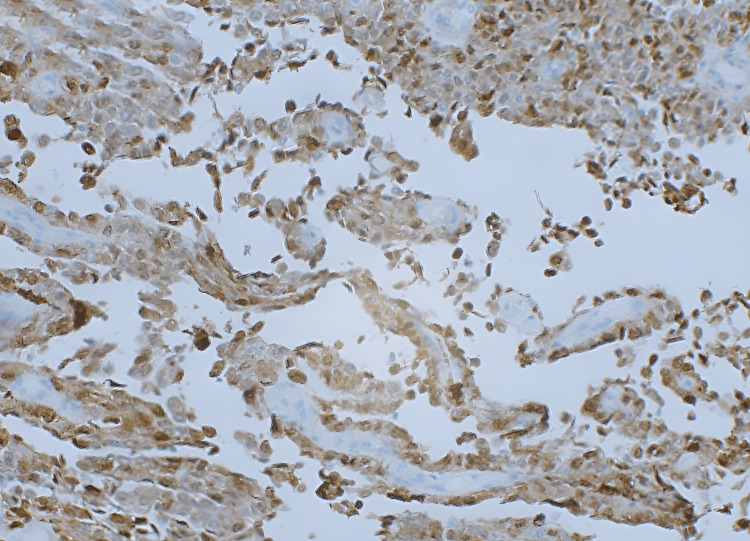
Immunohistochemical staining showed that S-100 was strongly and diffusely positive in both cytoplasmic and nuclear patterns.

Immunohistochemical staining demonstrated positivity for the S100 protein (Figure 3), while melanocytic markers were negative.

**Figure 4:**
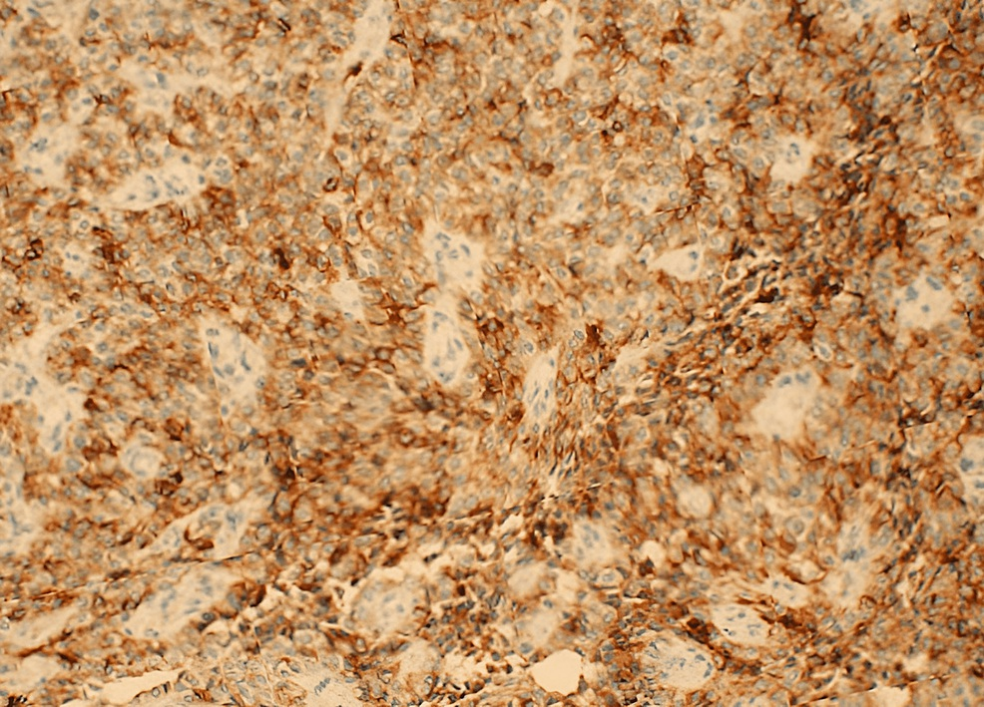
The tumor cells were positive for CD56.

Additionally, CD56 markers exhibited positive expression (Figure 4), further supporting the diagnosis of GNET.

To reduce the risk of disease recurrence, the patient received adjuvant chemotherapy consisting of a combination of doxorubicin and ifosfamide. Regular follow-up CT scans performed at three-month intervals demonstrated no evidence of disease recurrence. However, at the 12-month follow-up, the patient presented with symptoms indicative of intestinal obstruction, prompting a CT scan that revealed the presence of a recurrent tumor in the small intestine. Subsequently, a second surgical resection was performed to remove the recurrent tumor, followed by adjuvant chemotherapy involving etoposide and cisplatin. Remarkably, the patient remained free from disease during the subsequent 24 months of follow-up.

## Discussion

Malignant gastrointestinal neuroectodermal tumors (GNETs) are exceptionally rare neoplasms that originate from neural crest cells within the gastrointestinal tract. These tumors exhibit distinct histological and immunohistochemical characteristics, making them a unique entity within the spectrum of gastrointestinal malignancies.

The diagnosis of GNET poses considerable challenges due to its rarity and variability in its clinical presentation. Nonspecific symptoms such as abdominal pain, weight loss, and fatigue are commonly reported by patients. These symptoms, although suggestive, do not provide definitive evidence for GNET and can overlap with other gastrointestinal tumors, including gastrointestinal stromal tumors (GISTs), leiomyosarcomas, and carcinoid tumors.

Imaging studies, such as computed tomography (CT) scans and magnetic resonance imaging (MRI), play a crucial role in the diagnosis of GNET. These modalities aid in determining the location, size, and extent of the tumor. CT scans can reveal intraluminal masses with associated features such as bowel wall thickening and mesenteric lymphadenopathy. MRI can provide additional information regarding the relationship of the tumor with adjacent structures. These imaging techniques, while helpful, cannot definitively confirm the diagnosis and must be complemented with histopathological examination [5].

Histopathological examination, usually obtained through endoscopic biopsies or surgical resection, is essential for confirming the diagnosis of GNET. Microscopically, GNETs display a diverse range of histological patterns, including solid, nested, trabecular, and glandular formations. Immunohistochemical analysis is crucial in establishing the diagnosis, with GNETs typically exhibiting positivity for S100 protein and negativity for melanocytic markers. Additional markers, such as CD56 and synaptophysin, can further support the neural crest origin of these tumors [6].

The treatment of GNET remains challenging due to its rarity and the lack of established standard treatment guidelines. Surgical resection is considered the mainstay of treatment, with the extent of resection dependent on the tumor's location and size. Complete excision of the tumor is crucial to minimize the risk of local recurrence and improve patient outcomes. However, due to the aggressive nature of GNETs, they often infiltrate surrounding structures, making complete resection challenging [7].

Adjuvant chemotherapy has been explored as a potential treatment option for GNETs, but the optimal regimen and duration of treatment remain unclear. Some studies have suggested a benefit from adjuvant chemotherapy with ifosfamide and doxorubicin, but further research is needed to establish its efficacy [8].

The role of targeted therapies, such as tyrosine kinase inhibitors, in the treatment of GNETs is also being investigated, although limited data are available.

The prognosis of malignant GNETs is generally poor, with a high risk of recurrence and metastasis [2]. The rarity of these tumors limits our understanding of their natural history and optimal management strategies. Therefore, there is a crucial need for more research to establish standardized treatment guidelines, improve diagnostic accuracy, and explore novel therapeutic approaches for this rare and challenging tumor.

## Conclusions

A malignant gastrointestinal neuroectodermal tumor (GNET) is a rare tumor of the gastrointestinal tract that arises from the neural crest cells. The diagnosis of GNET is challenging due to its rarity and variable clinical presentation. Imaging studies and endoscopic biopsies can help in the diagnosis, and surgical resection is the mainstay of treatment. Adjuvant chemotherapy with ifosfamide and doxorubicin has shown some benefit, but the optimal regimen and duration of treatment are not well established. The prognosis of malignant GNET is generally poor, and there is a need for more research to establish standardized treatment guidelines for this rare tumor.
